# Resilience, well-being and informal and formal support in multi-problem families during the Covid-19 pandemic

**DOI:** 10.1186/s13034-022-00542-2

**Published:** 2022-12-19

**Authors:** Natasha Koper, Hanneke E. Creemers, Levi van Dam, Geert Jan J. M. Stams, Susan Branje

**Affiliations:** 1 Department of Youth and Family, Utrecht University, PO box 80140, 3508TC Utrecht, The Netherlands; 2 Department of Forensic Child and Youth Care Sciences, University of Amsterdam, Amsterdam, The Netherlands; 3 YIM Foundation, Amersfoort, The Netherlands; 4 Levvel Youth and Family Care, Amsterdam, The Netherlands

**Keywords:** Covid-19, Informal mentoring, Multi-problem families, Pandemic, Parents, Resilience, Support, Therapeutic alliance, Well-being, Youth

## Abstract

**Background:**

The Covid-19 pandemic may have had negative effects on youth and parental mental health, especially in high-risk populations such as multi-problem families (i.e., families that experience problems in multiple domains, such as mental health and social network problems). Using one to four assessments during all phases of the Covid-19 pandemic up until January 2022, we examined the associations between pandemic-related stress and mental health (resilience and well-being) of youth and parents from multi-problem families. We also investigated whether experienced informal (i.e., youth informal mentoring) and formal support (i.e., therapist support) served as protective factors in this association.

**Methods:**

A total of 92 youth aged 10–19 years (46.7% girls; mean age 16.00 years) and 78 parents (79.5% female; mean age 47.17 years) filled in one to four questionnaires between March 2020 and January 2022. Multi-level analyses were conducted to account for the nested structure of the data.

**Results:**

For youth, pandemic-related stress was associated with lower well-being, but not with resilience. Perceived support from both mentors and therapists was positively associated with youth mental health. Furthermore, high perceived therapist support protected youth from the negative effect of pandemic-related stress on resilience. For parents, pandemic-related stress was not related to mental health, irrespective of therapist support. Yet, therapist support was directly and positively associated with parental mental health.

**Conclusions:**

Youth from multi-problem families who experience pandemic-related stress are at risk of (elevated) mental health problems during the pandemic, specifically if they have no or weak therapist support. The mental health of parents, however, was minimally affected by pandemic-related stress, indicating strength and flexibility. Youth and parents who experienced support during the pandemic reported higher levels of resilience and well-being, demonstrating the importance of support for individuals’ mental health during stressful times such as a pandemic.

**Supplementary Information:**

The online version contains supplementary material available at 10.1186/s13034-022-00542-2.

The coronavirus (Covid-19) pandemic pushed governments all over the world to take extraordinary and severe measures to fight the virus. Despite the effectiveness of measures such as lockdowns and physical distancing to restrain the spreading of the virus, there may have been be a negative impact of the pandemic and related measures on individuals’ mental health [1, 2]. As a result of the imposed restrictions, many youth and their parents were forced to spend most of their time at home and less time with extended family and friends, potentially limiting the possibilities for support from their informal (i.e., social) and formal (i.e., professional) networks.

Results of studies on the impact of the Covid-19 pandemic on youth are heterogeneous, and suggest that for a sizable group (but not for everyone) the imposed restrictions during the pandemic negatively affected youth mental health [1, 2], mediated by increased stress [3]. People who already were vulnerable before the pandemic tended to suffer more [4, 5]. Yet, research on the mental health of multi-problem families, i.e., families who experience problems on several life domains, including mental health and social network problems [6, 7], during the Covid-19 pandemic seems lacking.

## Mental health during the Covid-19 pandemic

Both youth and parents have likely been affected by the imposed measures [8, 9], such as school closure and working from home, as these measures caused shifts in family routines, daily functioning and social connectedness [10]. Since the start of the Covid-19 pandemic, there has been continued interest in monitoring individuals’ mental health changes. Resilience and well-being are two important and relevant factors of mental health during a pandemic. Resilience is the capacity to cope with adversity and protects individuals from negative consequences of stressful events [11, 12], including the Covid-19 pandemic [13, 14]. Well-being refers to individuals’ subjective, psychological well-being, and is an important general indicator for mental health [15]. Some studies indeed demonstrated that the pandemic negatively affected youth and parents’ well-being [2, 16–19].

### Differential impact of the Covid-19 pandemic on mental health

The pandemic seems to not have affected all youth and parents equally. That is, whereas some families experienced difficulties adjusting during the pandemic, others were able to cope relatively well [5], suggesting that some were more resilient than others. Several risk and resilience factors might explain why some families are more severely impacted by the pandemic than others [5, 20]. Prepandemic risk factors, such as low socioeconomic status and mental health problems, seem to exacerbate the effects of the pandemic, placing already vulnerable families at even greater risk of experiencing stress and low mental health during the pandemic [4, 21]. Multi-problem families face several problems in multiple domains, such as psychosocial functioning, family functioning, mental health, financial situation and functioning in their social networks. These problems are often chronic and intergenerational [6, 7]. As a result, youth and parents from multi-problem families may be at increased risk for negative effects of the pandemic on their mental health.

Another factor that exacerbates the effects of the pandemic is perceived pandemic-related stress [3, 16]. Pandemic-related stress is the experienced stress as a result of the pandemic, both due to the virus itself and imposed restrictions. For instance, people may experience pandemic-related stress due to isolation [1], unpredictability and daily routine disruptions, increased exposure to information about threats to well-being [10], and illness, unexpected loss and grief [22]. Pandemic-related stress subsequently negatively affects mental health [3, 16, 23]. Given the pre-existing risk factors in multi-problem families [6, 7], these families are more likely to experience pandemic-related stress [4, 21].

### Support as protective factor during adversity

Support from friends, relatives and professionals can protect individuals from developing problems in stressful situations [24], and is therefore considered an important protective factor against the impact of the Covid-19 pandemic [2, 25]. Support can be offered by many different individuals from one’s social network, which can be broadly divided into the informal (i.e., natural) and the formal (i.e., professional) network. Informal networks consist of friends, family members, acquaintances and others with whom individuals have organically formed relationships. The informal network may also include nonparental adults who provide youth with support by offering help and advice, thereby promoting their mental health (e.g., 26–28). The benefits of these mentoring relationships can last into adulthood, even for youth who experienced childhood adversities [29], and preliminary evidence suggests that mentors may also play a crucial role in offering support to youth during the Covid-19 pandemic [30]. Particularly when the perceived relationship quality is high, youth are likely to experience benefits from informal mentors [26, 31].

Formal networks consist of professionals who are involved in the lives of youth and parents, such as teachers, counsellors, and therapists. Given that mental health needs may have increased as a result of the pandemic [17, 32], therapists may play an important role in offering support to families during the Covid-19 pandemic. As for informal support, perceived relationship quality, or therapeutic alliance, seems to determine the effectiveness of formal support [33, 34]. However, the pandemic has led to therapy disruptions [35] and changes in therapy delivery from physical appointments to videoconferencing or telephone consultations, which negatively affected the therapeutic alliance for some clients [32, 36].

In sum, there is evidence suggesting that experienced support from informal mentors and therapists can protect youth and parents from (elevated) mental health problems, and may buffer against the negative impact of stress resulting from the Covid-19 pandemic. Multi-problem families tend to have unstable informal networks [37] and often experience interrupted and fragmented formal support [38], making it more likely that they experience low levels of support, increasing the risk for mental health problems, especially during a pandemic. Therefore, it is particularly important to examine whether support can protect youth and parents from mental health problems during the Covid-19 pandemic.

### Present study

In this study we examined the associations between pandemic-related stress, support and mental health in multi-problem families during the Covid-19 pandemic. More specifically, we tested three hypotheses: (1) pandemic-related stress is negatively associated with youth and parental mental health; (2) support is positively associated with youth and parental mental health; and (3) support is a protective factor minimizing the negative effects of pandemic-related stress on youth and parental mental health. We aimed to give deeper insight into mental health and the functioning of support structures in the vulnerable population of multi-problem families during the pandemic. To meet this aim, we performed multi-level regression analyses and included several covariates to control for the potential confounding influence on youth and parents’ mental health: demographics, treatment duration, and treatment condition. We also included pandemic duration and severity level of imposed pandemic measures as predictors of mental health. This study was preregistered at OSF Registries (osf.io/z7wvr).

## Methods

### Procedure

Part﻿icipants were multi-problem families receiving youth and family care who participated in a quasi-experimental multi-site study called *Growth in personal environment* (GRIP) [39]. The GRIP study is registered at the Netherlands Trial Register (NL7565). The design of the study is in accordance with the guidelines of Helsinki (1964) and its later amendments, and approved by the faculty ethical review board of the Faculty of Social and Behavioral Sciences of Utrecht University (FETC-18-093). The current study is preregistered at OSF Registries (osf.io/z7wvr).

Data for GRIP were collected from December 2019 to January 2022 at five organizations for youth and family care located in urban areas in the Netherlands. The aim of the GRIP study is to investigate the effectiveness of the InConnection approach, an outreaching, systemic approach for multi-problem families in which youth nominate an informal mentor according to the Youth-Initiated Mentoring (YIM) approach [40, 41]. The effects are compared with a control group, which received care as usual including several multi-modal outpatient systemic treatment programs for multi-problem families without YIM (for more information on the conditions, see [39]).

Families that started treatment in one of the treatment groups in the GRIP study were informed about this study by an employee of the care providing organization, often the case manager. The employee asked verbal permission from the client system to share their contact details with the independent research team. A member of the research team then contacted the client system, informed them of the study, and suggested to schedule an appointment. Active informed consent for participation in the GRIP study was received from youth and parents for their own participation. For youth under the age of 16, active informed consent for their participation was also obtained from one parent or guardian [39].

The GRIP study aimed to assess changes in outcomes during youth and family care by using four multi-informant assessments including questionnaires: (1) at the start of treatment; (2) after 3 months; (3) after 9 months; and (4) after 15 months. At the first assessment, the youth and parents completed questionnaires at a chosen location, often at home, in the presence of a member of the research team who assisted participants in answering the questions if problems, such as reading problems, were present. If the participant did not experience problems in answering the questions, subsequent assessments were completed independently online. To comply with the measures against the coronavirus taken by the Dutch government, we temporarily replaced home visits by phone and video calls during various phases of the pandemic. Participants received a financial reward of €50 for completion of the four assessments. For this study, we used all available assessments during the pandemic per participant. We set the starting date of the Covid-19 pandemic in the Netherlands at March 23, 2020, which was when the first lockdown was announced by the Dutch government.

### Participants

Families were approached for participation in this study if: (1) families consisted of at least one youth aged 10 to 23 years; (2) families experienced problems, such as school drop-out, divorce, trauma, antisocial behavior, and substance use, that are considered complex, multiple and severe, and received indicated intensive treatment from specialized youth and family care organizations for these problems; (3) previous treatments have not yielded the intended effects, and/or youth have an indication for an out-of-home placement; 4) families had sufficient Dutch proficiency.

The GRIP study included 102 youth and 86 parents, of which 92 youth and 78 parents were selected for the current study, because they completed one to four assessments during the pandemic. Of these youth, 59 were in the intervention group (64.1%), and the remaining 33 in the control group (35.9%). For the current study, both treatment groups were combined. Mean age of the youth was 16.00 years at the start of the pandemic (*SD* = 1.73, range = 10.59–19.19 years), and 43 were girls (46.7%). Most youth were attending school at the first measurement occasion during the Covid-19 pandemic (87.0%), and more than half followed preparatory secondary vocational education (59.8%). Most youth identified as Dutch (73.9%) or partly Dutch (5.4%); the others identified as Surinam (3.3%), Antillean (1.1%), or other (12.0%). At the start of the pandemic, 42 youth lived with their parents: 27 lived with one of their parents or alternately with either parent (29.3%), and 15 lived with both parents (16.3%). Three lived by themselves (3.3%), 31 lived in a residential facility (33.8%), six with friends or family (6.5%), and six in a foster home (6.5%). Most youth received youth and family care for the entire duration of the pandemic (78.2%). Three youth did not receive any care during the pandemic (3.3%). The others received care for some time during the pandemic (18.5%). Most youth had an informal mentor (56.5%) during the pandemic, which was most often a family member (44.2%).

Of the 78 participating parents, biological parents participated most often (85.9%), and adoptive parents, foster parents and stepparents were less common (14.1%). In most families one parent participated (74.4% of parents), in the remaining 10 families, two parents participated (25.6% of parents), which were mostly two biological parents. Forty-six parents were in the intervention group (59.0%), and the remaining 32 were in the control group (41.0%). On average, parents were 47.17 years old at the start of the pandemic (*SD* = 7.33, range = 28.84–64.35) and 62 parents were female (79.5%). Most parents were married or living together with a partner (45.5%), 20 were divorced or separated (26.0%), 20 were unmarried (26.0%), and two were widowed (2.6%). Most parents lived with children (84.0%), and identified as Dutch (90.9%) or partly Dutch (1.3%), the others identified as Surinam (2.6%), Antillean (1.3%), or other (3.9%). Five parents finished no formal education or primary education only (6.6%), 22 finished secondary education (28.9%), 16 finished vocational education (21.1%), 26 finished higher education (34.2%), and 7 finished another type of education (9.2%). For the majority of parents the net monthly income (NMI) was in the lowest 10% of Dutch adults [42]: 29 parents (41.4%) had a NMI of less than €1.600, and 17 parents (24.3%) had a NMI of €1.601-€2.100. Most parents received youth and family care for the entire duration of the pandemic (69.2%) or for some time during the pandemic (20.5%), and some parents did not receive care at any time point during the pandemic (10.3%).

### Missing data

On average, youth and parents reported on two measurements during the pandemic (*n*_*youth*_ = 23 and *n*_*parents*_ = 22 on one measurement, *n*_*youth*_ = 29 and *n*_*parents*_ = 25 on two measurements, *n*_*youth*_ = 12 and *n*_*parents*_ = 8 on three measurements, and *n*_*youth*_ = 28 and *n*_*parents*_ = 23 on four measurements). Thus, non-completion was high: 69.6% for youth and 70.5% for parents. Non-completion was most often due to the design of the GRIP study: Participants filled out questionnaires four times and many had already been completed before the pandemic started. Non-completion due to design occurred in 45 cases in youth (48.9%) and 42 cases in parents (53.8%). Logistic regression revealed two differences in demographics between completers and non-completers: Youth differed in living situation (*p* = 0.037), indicating that completers were more likely to be living elsewhere than with their parents. Parents differed on ethnic identity (*p* = 0.020), showing that non-completers were more likely to identify as Dutch. Youth and parents who completed all four measurements did not differ from non-completers on other demographic variables (i.e., age, gender, ethnic identity, living situation, and going to school; *p*s > 0.134 for youth and *p*s > 0.383 for parents).

Missing data of study variables were also analyzed on item level. Little’s missing completely at random (MCAR) test [43] showed that data was missing completely at random, χ^2^(31) = 37.70, *p* = 0.190 for youth, and χ^2^(44) = 50.33, *p* = 0.237 for parents. Hence, all participants were included in the analyses to allow all available data to be used.

### Measurements

#### Resilience

Resilience of youth and parents, defined as the capacity of the individual and its social and physical environment to cope with adversity [11], was measured at all assessments by age-appropriate self-reported resilience measures. Youth filled in the Child and Youth Resilience Measure-Short form (CYRM-12) and parents filled in the Adult Resilience Measure-Short form (ARM-12), both consisting 12 items [44–46]. Both versions assess the resources (individual, relational, communal and cultural) available to individuals that may sustain their resilience (e.g., “I know where to go in my community to get help” and “My family will stand by me during difficult times”). Items are rated on a 5-point scale from 1 = *does not describe me at all* to 5 = *describes me a lot*. To establish a score for resilience, a mean score is calculated using the 12 items of the CYRM-12 and ARM-12 [44–46], for youth and parents respectively. Higher scores reflect higher levels of resilience. Internal consistency of the CYRM-12 was satisfactory in the original Canadian sample [44] and a Dutch sample [47] (α = 0.84 and α = 0.93, respectively). The CYRM-12 showed sufficient content validity to be used as a cross-cultural screener of resilience [44]. In contrast to the CYRM-12, psychometric properties of the ARM-12 have not been examined yet. The internal consistencies were good in the current samples (α = 0.82 for youth and α = 0.81 for parents).

#### Well-being

Youth and parental well-being was measured at each assessment using the self-reported World Health Organization Well-Being Index (WHO-5), which assesses subjective psychological well-being [48]. Youth and parents rated five items (e.g., “I have felt cheerful and in good spirits” and “I woke up feeling fresh and rested”) on a 6-point scale from 0 = *none of the time* to 5 = *all the time*. To establish a score for well-being, we calculated the mean score of the five items of the WHO-5. Higher scores reflect higher levels of well-being. The internal consistency and validity were satisfactory in a variety of samples [15], including a Dutch sample (α = 0.91–0.93) [49]. The internal consistencies were good in the current samples (α = 0.89 for youth and α = 0.87 for parents).

#### Pandemic-related stress

Experienced stress related to the Covid-19 pandemic by youth and parents was measured at each assessment using 12 or 11 statements, respectively. The items tap into different potential stressors during the pandemic, including health concerns, financial problems, and relationship and social issues (e.g., “The coronavirus crisis leads to money problems for me and/or my family” and “Due to the coronavirus crisis, I often argue with my family members”). The youth version contains an extra item concerning education (“I am afraid that my education will be delayed due to the coronavirus crisis”). See Additional file 1: Table S1 for all items of this questionnaire. Both youth and parents rated the items using a 5-point scale ranging from 1 = *totally disagree* to 5 = *totally agree*. A score for pandemic-related stress was calculated using a mean score after recoding positively phrased items. Higher scores reflect more pandemic-related stress. The internal consistencies were adequate in the current samples (α = 0.79 for both youth and parents).

#### Informal support

Informal support was measured in youth and operationalized as the support from an informal mentor, which is an older or more experienced individual from the youth’s informal network [40]. Two variables were created: a dichotomous variable indicating the presence of an informal mentor (mentor/no mentor), and a continuous variable for perceived informal support, reflecting the quality of the relationship with the informal mentor. For perceived informal support, youth completed the Psychological Availability and Reliance on Adult (PARA) questionnaire, which is designed to measure relationship quality in asymmetrical relationships such as mentoring relationships from an attachment perspective. It measures three aspects of the relationship: availability, reliance, and affective bond (e.g., “You go to your informal mentor for support or advice” and “Your informal mentor listens to you in a sympathetic manner”) [50, 51]. Two items of the original affectional bond scale were deleted, as they were not deemed appropriate for the informal mentoring relationship (e.g., “You dread knowing you may have another informal mentor in the future”), resulting in a 17-item scale. Youth rated items on a 4-point scale from 1 = *disagree* to 4 = *agree*. To establish a score for perceived informal support, mean scores were calculated based on the 17 items after recoding negatively phrased items. Participants who did not have an informal mentor at the time of the assessment did not fill out the PARA and received a score of 1, which is the lowest possible score. Higher scores reflect higher levels of perceived informal support. The internal consistency (α = 0.65–0.81) and validity were satisfactory for most scales of the PARA in a Dutch sample [50]. The internal consistency was examined based on scores of youth with informal mentors, and was good in the current sample (α = 0.89).

#### Formal support

Formal support was operationalized as the support from a therapist youth and parents experienced. Two variables were created: a dichotomous variable indicating the presence of a therapist (therapist/no therapist), and a continuous variable reflecting perceived formal support, that is, the therapeutic alliance. For perceived formal support, parents completed the Session Rating Scale (SRS), a four-item measure of therapeutic alliance, and youth completed the age-appropriate Child Session Rating Scale (CSRS). The (C)SRS taps into the relational bond between the therapist and client, agreement on the goals of therapy, agreement on the tasks of therapy, and the client’s view of the sessions (e.g., “I felt heard, understood, and respected” for the SRS and “The therapist listened to me” for the CSRS) [52]. Both youth and parents rated the items on a visual analogue scale of 10 cm, where the left side indicates a more negative response and the right side indicates a more positive response. To establish a score for perceived formal support, mean scores were calculated based on the four items, resulting in a possible range of 1–10. Participants who did not receive treatment from one of the teams participating in the GRIP study [39] at the time of the assessment did not fill out the (C)SRS and were scored 1, which is the lowest possible score. Higher scores reflect higher satisfaction with formal support. The internal consistencies (α = 0.85–0.95) and validity of the SRS were satisfactory to good in Dutch samples [53]. The internal consistencies were adequate to good in the current samples (α = 0.95 for youth and α = 0.94 for parents).

#### Covariates

Several covariates were measured to control for potential confounding variables in the analyses: demographics, treatment duration, and treatment condition. We also included pandemic duration and severity level of imposed pandemic measures as predictors of mental health.

Background information regarding youth and parents was obtained with a basic demographics and family functioning form completed at each assessment. This form also included information on whether treatment was still offered to the families and whether youth were going to school at the time of the assessment. Demographics that were tested as covariates, were: age, gender (male/female), ethnic identity (Dutch/non-Dutch), living situation (for youth: with parents/elsewhere; for parents: with children/without children), and going to school (yes/no; for youth only).

Treatment duration was calculated to control for differences between participants receiving treatment. We calculated how many days the treatment endured at each assessment. If participants had already finished treatment at the time of the assessment, we included the total number of days the treatment had lasted for. Treatment condition was included to control for differences between the intervention and control groups.

Covid-19 pandemic duration was calculated to control for differences between individuals in duration of the pandemic at each assessment. We calculated how many days after the start of the pandemic (March 23, 2020) assessments took place.

Covid-19 pandemic measures severity level was determined to control for differences in the severity of measures between participants at each assessment. We established a severity level following the pandemic strategy of the Dutch government; four levels were specified based on the level of risk: 1 = *vigilant*, 2 = *worrisome*, 3 = *serious*, and 4 = *very serious*. Table 1 provides information on how the risk levels are determined. See Additional file 1: Table S2 for a summary of the active measures during each risk level.

**Table 1:** Determination of risk levels by the Dutch Government

	1. Vigilant	2. Worrisome	3. Serious	4. Very serious
Positive tests per 100.000 inhabitants per week	< 35	35–100	100–250	> 250
Hospital admissions (incl. IC) per 1.000.000 inhabitants per week	< 4	4–16	16–27	> 27

Adapted from https://coronadashboard.rijksoverheid.nl/over-risiconiveaus. Copyright 2021 by Central Government of the Netherlands

### Statistical analyses

All analyses were performed in Mplus 8.7 [54]. Descriptive statistics were obtained to gain insight in the means and standard deviations of the variables, and univariate associations between each pair of variables. All continuous variables were centered to allow for interaction variables to be created. We created interaction variables using pandemic-related stress and the continuous informal and formal support variables.

We performed multilevel regression analysis (also referred to as hierarchical linear models) to account for the nested structure of our data. More specifically, two-level models were examined in which assessments (Level 1) were nested within participants (Level 2). Intraclass correlations (ICC) at Level 2 were 0.58 for youth resilience, 0.29 for youth well-being, 0.76 for parental resilience, and 0.36 for parental well-being.

We performed three sets of regression analyses. In Model 1, we added all potential covariates into the model to examine which were significantly related to the outcome. In the subsequent models we included only the significant covariates to create more parsimonious models. In Model 2, we tested whether pandemic-related stress (*hypothesis 1*) and informal and formal support (*hypothesis 2*) were related to resilience and well-being during the pandemic. That is, we examined whether individuals reported lower resilience and well-being at times when they reported more pandemic-related stress. We added both the dichotomous and continuous support variables in these analyses. In Model 3, we tested whether informal and formal support moderated the link between pandemic-related stress and resilience and well-being (*hypothesis 3*). These analyses were also conducted with both the continuous and dichotomous support variables. We performed separate analyses for youth and parents, for resilience and well-being, and for informal and formal support, resulting in six regression analyses (the analyses including informal support were performed for youth only).

By interpreting the results at Level 1, we looked at within-person correlated change. We used the *p* < 0.05 criterion to determine the significance of the effects. The effect sizes of models are reported using explained variance (*R*^2^ values); 0.02 was considered small, 0.13 medium and 0.26 large [55]. Full information maximum likelihood estimation was used to deal with observations with incomplete or missing data. All models were saturated and therefore had a perfect fit, thus, fit statistics are not reported.

## Results

### Descriptive statistics

Descriptive statistics of youth and parental resilience, well-being, pandemic-related stress, and informal and formal support are shown in Table 2. Associations between all variables including covariates are presented in Table 3.

**Table 2:** Descriptive statistics of youth and parental resilience, well-being, pandemic-related stress, informal and formal support, and covariates

	Youth			Parents		
	*M* (*SD*)	Range	%	*M* (*SD*)	Range	%
Resilience	3.69 (0.55)	1.84–4.83		4.02 (0.50)	2.67–4.92	
Well-being	2.75 (1.04)	0.20–4.60		2.90 (0.86)	0.70–5.00	
Pandemic-related stress	2.36 (0.63)	1.17–3.92		2.37 (0.64)	1.18–4.07	
Informal support	3.30 (0.46)	2.12–4.00		–	–	
Formal support	6.71 (2.04)	1.00–10.00		7.63 (1.82)	1.50–10.00	
Pandemic duration in days	229.61 (177.62)	1–674		231.11 (175.00)	1–634	
Pandemic measures severity level (*vigilant*)			26.6%			24.5%
Pandemic measures severity level (*worrisome*)			17.9%			16.0%
Pandemic measures severity level (*serious*)			23.1%			27.7%
Treatment duration in days	284.13 (164.01)	–13 to 710		288.31 (157.34)	15–710	
Treatment condition (*intervention group*)			64.1%			59.0%

Means are calculated per person across assessments. Percentages are calculated per assessment (if applicable). The statistics for support are based on individuals who have reported on the experienced support, thus, excluding individuals without mentors or therapists. Since some participants filled out the first questionnaire prior to starting treatment, the range of treatment duration varies from a negative to a positive number of days

**Table 3:** Associations (β) between youth and parental resilience, well-being, pandemic-related stress, informal and formal support, and covariates

		1	2	3	4	5	6	7	8	9	10	11	12	13	14	15
1	Resilience	–	0.29*	− 0.31*	–	–	0.05	0.48*	0.15	0.18	0.02	0.00	0.02	− 0.04	− 0.02	− 0.02
2	Well-being	0.68**	–	− 0.24	–	–	0.08	0.23	0.12	− 0.04	0.13	− 0.04	− 0.13	0.00	− 0.10	− 0.08
3	Pandemic-related stress	− .0.11	− .0.22*	–	–	–	− 0.10	− 0.13	− 0.23	− 0.05	0.13	− 0.27*	− 0.08	0.26*	− 0.02	− 0.08
4	Mentor presence	-0.23*	− 0.07	− 0.12	–	–	–	–	–	–	–	–	–	–	–	–
5	Mentor relationship quality	0.50**	0.38*	0.18	–	–	–	–	–	–	–	–	–	–	–	–
6	Therapist presence	− 0.11	0.01	− 0.12	0.25**	0.10	–	–	0.02	− 0.01	− 0.23**	0.11	0.38**	0.13	0.09	0.00
7	Therapeutic alliance	0.51**	0.37**	0.24*	− 0.23*	0.37*	–	–	.09	− 0.12	− 0.17	− 0.54	0.04	− 0.16	0.13	0.16
8	Age	.07	− .01	− .05	− .01	.19*	− .29	.25	–	− .08	− .07	.29*	− .02	.81	.17	− .36**
9	Gender	− 0.14	− 0.36*	0.19	− 0.17	0.22	− 0.70	0.02	0.19*	–	0.09	0.19*	0.21	− 0.65	− 0.16	− 0.05
10	Ethnic identity	− 0.16	0.12	− 0.14	0.12	− 0.13	0.06	− 0.15	0.03	− 0.08	–	− 0.15**	0.31	0.30	− 0.12	− 0.30**
11	Living situation	− .032*	− 0.42**	0.23*	− 0.17	0.00	− 0.09	− 0.09	0.26	0.13	0.10	–	0.12	0.11	0.07	− 0.06
12	Pandemic duration	− 0.06	− 0.07	− 0.01	0.21*	− 0.08	0.25**	− 0.06	− 0.79	− 0.67	0.07	0.04	–	− 0.14*	0.92**	− 0.44*
13	Pandemic measures severity level	− 0.01	− 0.03	0.10	− 0.01	0.04	− 0.05	− 0.01	− 0.04	0.77	0.46	− 0.02	− 0.14*	–	− 0.09	0.03
14	Treatment duration	− 0.01	− 0.03	0.03	0.17	− 0.00	–	− 0.02	− 0.62	− 0.59	− 0.34	− 0.09	0.97**	− 0.03	–	− 0.13
15	Treatment condition	0.41**	0.29*	− 0.06	− 0.38*	0.09	− 0.08	0.38*	− 0.01	0.08	0.00	0.01	− 0.51	0.04	− 0.26	–

Associations for the youth sample (*n* = 92) are shown below the diagonal. Associations for the parent sample (*n* = 78) are shown above the diagonal. The statistics for mentor relationship quality and therapeutic alliance are based on individuals who have reported on the experienced support, thus excluding individuals without mentors or therapists. Irrelevant associations have been left out (e.g., between mentor presence and mentor relationship quality). The covariate going to school has been dropped due to insufficient variation

^*^p < .05. **p < .001

### Model 1: covariates

#### Youth data

Analyses on youth data showed that gender, treatment condition, and living situation were significant covariates of resilience and/or well-being. Boys (*M* = 3.05, *SD* = 0.95) had higher scores on well-being than girls (*M* = 2.41, *SD* = 1.05), β = - 0.35, *SE* = 0.13, *p* = 0.009. Youth in the intervention group had higher scores on resilience and well-being (*M* = 3.84, *SD* = 0.50, and *M* = 2.93, *SD* = 1.02, respectively) than youth in the control group (*M* = 3.42, *SD* = 0.53, and *M* = 2.43, *SD* = 1.01, respectively), β = 0.37, *SE* = 0.10, *p* < 0.001, and β = 0.25, *SE* = 0.11, *p* = 0.024, respectively. Youth living with their parents had higher scores on resilience and well-being (*M* = 3.79, *SD* = 0.61, and *M* = 2.96, *SD* = 1.16, respectively) than youth who lived elsewhere (*M* = 3.57, *SD* = 0.60, and *M* = 2.46, *SD* = 1.18, respectively), β = − 0.28, *SE* = 0.12, *p* = 0.015, and β = − 0.31, *SE* = 0.11, *p* = 0.006, respectively. Therefore, gender, treatment condition, and living situation were included as covariates in subsequent analyses. Age, ethnic identity, pandemic duration, pandemic severity level and treatment duration were not significant and are thus left out. Youth went to school at almost all of the assessments during the pandemic (91.6%). Therefore, we could not reliably estimate the influence of this covariate and dropped it.

#### Parent data

Analyses on parent data showed that none of the covariates were significant. Therefore, no covariates were added to the subsequent analyses with parent data.

### Model 2: Predictors of resilience and well-being

#### Youth resilience

In Model 2a we examined pandemic-related stress and informal support as predictors of youth resilience. Results showed that pandemic-related stress was not significantly related to youth resilience, β = −0.11, *SE* = 0.11, *p* = 0.330. Informal support, however, was significantly related to resilience: Having a mentor was positively associated with resilience, β = 0.92, *SE* = 0.20, *p* < 0.001, and higher levels of mentor relationship quality were associated with higher levels of resilience, β = 1.14, *SE* = 0.20, *p* < 0.001. The within-effects of this model were medium in size, *R*^*2*^ = 0.21.

Next, in Model 2b we examined pandemic-related stress and formal support as predictors of resilience. Again, results showed that pandemic-related stress was not significantly related to youth resilience, β = −0.14, *SE* = 0.11, *p* = 0.195. In addition, the presence of a therapist was not a significant predictor of youth resilience, β = 0.11, *SE* = 0.15, *p* = 0.456, but higher levels of therapeutic alliance were associated with higher levels of resilience, β = 0.53, *SE* = 0.13, *p* < 0.001. The within-effects of this model were medium in size, *R*^*2*^ = 0.25.

#### Youth well-being

In Model 2a we examined pandemic-related stress and informal support as predictors of youth well-being. Results showed that at times when youth reported higher levels of pandemic-related stress, they reported lower levels of well-being, β = −0.18, *SE* = 0.09, *p* = 0.035. Additionally, informal support was positively associated with youth well-being: Having a mentor significantly predicted well-being, β = 0.70, *SE* = 0.34, *p* = 0.040, and higher levels of mentor relationship quality were related to higher levels of well-being, β = 0.84, *SE* = 0.34, *p* = 0.015. The within-effects of this model were medium in size, *R*^*2*^ = 0.18.

In Model 2b we examined pandemic-related stress and formal support as predictors of youth well-being. Again, results showed that higher levels of pandemic-related stress were related to lower levels of youth well-being, β = −0.20, *SE* = 0.09, *p* = 0.030. The presence of a therapist was not significantly related to youth well-being, β = 0.16, *SE* = 0.15, *p* = 0.268. Yet, higher levels of therapeutic alliance were associated with higher levels of youth well-being, β = 0.33, *SE* = 0.14, *p* = 0.022. The within-effects of this model were medium in size, *R*^*2*^ = 0.16.

#### Parental resilience

In Model 2 we examined pandemic-related stress and formal support as predictors of parental resilience. Results showed that pandemic-related stress was not associated with parental resilience, β = −0.25, *SE* = 0.21, *p* = 0.237. Formal support, however, was positively associated with parental resilience: Receiving treatment was associated with resilience, β = 0.57, *SE* = 0.22, *p* = 0.008, and higher levels of therapeutic alliance predicted higher levels of parental resilience, β = 0.58, *SE* = 0.27, *p* = 0.030. The within-effects of this model were medium in size, *R*^*2*^ = 0.14.

#### Parental well-being

In Model 2 we examined pandemic-related stress and formal support as predictors of parental well-being. Results showed that pandemic-related stress was not significantly related to parental well-being, β = −0.19, *SE* = 0.12, *p* = 0.118. Formal support, however, was a significant predictor of parental well-being: Receiving treatment was associated with well-being, β = 0.56, *SE* = 0.21, *p* = 0.008, and higher levels of therapeutic alliance were related to higher levels of well-being, β = 0.58, *SE* = 0.21, *p* = 0.007. The within-effects of this model were small in size, *R*^*2*^ = 0.12.

#### Sensitivity analyses

We conducted sensitivity analyses to check the robustness of our results of the perceived support variables as predictors of resilience and well-being (Model 2). In these analyses we excluded participants without mentors or therapists. The results (available upon request) were very similar to the initial analyses, giving us confidence in the accuracy of our initial results.

### Model 3: Interactions between pandemic-related stress and support

**Fig. 1:**
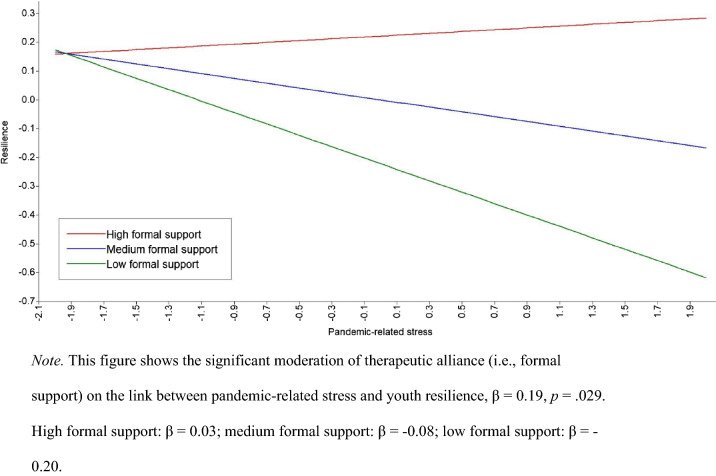
Interaction of pandemic-related stress and therapeutic alliance as predictor of youth resilience

In six separate models, we tested the interaction effects between pandemic-relation stress and perceived support on resilience and well-being, to examine whether the associations between pandemic-related stress and mental health are affected by perceived support. Just one of these interaction effects was significant: The interaction between pandemic-related stress and therapeutic alliance was a significant predictor of youth resilience, β = 0.19, *SE* = 0.09, *p* = 0.029. Inspection of Fig. 1 reveals that for youth who experience no or low levels (-1 *SD*) of therapeutic alliance, pandemic-related stress is negatively related to resilience, *B* = −0.12, *SE* = 0.09, *p* = 0.036. For youth with average (*M*) and high levels (+1 *SD*) of therapeutic alliance, however, there is no significant link between pandemic-related stress and resilience, *B* = −0.08, *SE* = 0.06, *p* = 0.164, and *B* = 0.03, *SE* = 0.06, *p* = 0.595, respectively. The within-effects of this model were large in size, *R*^*2*^ = 0.29. Results of the models with interaction effects are presented in Table 4 (youth data) and Table 5 (parent data).

**Table 4:** Results of the models predicting youth resilience and well-being (n = 92)

Outcome variable	Resilience				Well-being			
Support type	Informal support		Formal support		Informal support		Formal support	
	β (*SE*)	*p*	β (*SE*)	*p*	β (*SE*)	*p*	β (*SE*)	*p*
Model 2								
Pandemic-related stress	− 0.11 (0.11)	.330	− 0.14 (0.11)	.195	− 0.18 (0.09)	.035	− 0.20 (0.09)	.030
Support (dichotomous)	0.92 (0.20)	.000	0.11 (0.15)	.456	0.70 (0.34)	.040	0.16 (0.15)	.268
Support (continuous)	1.14 (0.20)	.000	0.53 (0.13)	.000	0.84 (0.24)	.015	0.33 (0.14)	.022
Living situation	− 0.25 (0.10)	.011	− 0.24 (0.10)	.016	− 0.27 (0.11)	.017	− 0.27 (0.11)	.019
Treatment condition	0.35 (0.12)	.003	0.36 (0.12)	.002	0.27 (0.12)	.019	0.26 (0.12)	.033
Gender	–	–	–	–	− 0.43 (0.12)	.000	− 0.40 (0.13)	.002
Model 3								
Pandemic-related stress	− 0.11 (0.11)	.333	− 0.14 (0.10)	.174	− 0.18 (0.09)	.035	− 0.20 (0.09)	.029
Support (dichotomous)	0.90 (0.20)	.000	0.08 (0.15)	.598	0.70 (0.34)	.040	0.16 (0.14)	.270
Support (continuous)	1.11 (0.19)	.000	0.52 (0.13)	.000	0.84 (0.35)	.015	0.33 (0.15)	.022
Stress × Support (continuous)	0.06 (0.07)	.413	0.19 (0.09)	.029	− 0.01 (0.07)	.934	0.01 (0.07)	.863
Living situation	− 0.25 (0.10)	.013	− 0.24 (0.10)	.017	− 0.27 (0.11)	.016	− 0.27 (0.11)	.020
Treatment condition	0.35 (0.11)	.002	0.36 (0.11)	.001	0.27 (0.12)	.019	0.26 (0.12)	.032
Gender	–	–	–	–	− 0.41 (0.13)	.001	− 0.40 (0.13)	.002

**Table 5:** Results of the models predicting parental resilience and well-being (n = 78)

Outcome variable	Resilience		Well-being	
	β (*SE*)	*p*	β (*SE*)	*p*
Model 2				
Pandemic-related stress	− 0.25 (0.21)	.237	− 0.19 (0.12)	.118
Formal support (dichotomous)	0.57 (0.22)	.008	0.56 (0.21)	.008
Formal support (continuous)	0.58 (0.27)	.030	0.58 (0.21)	.007
Model 3				
Pandemic-related stress	− 0.24 (0.21)	.242	− 0.20 (0.12)	.088
Formal support (dichotomous)	0.57 (0.22)	.011	0.58 (0.22)	.009
Formal support (continuous)	0.58 (0.27)	.032	0.58 (0.23)	.010
Stress × Support (continuous)	0.02 (0.17)	.921	− 0.15 (0.11)	.160

## Discussion

This study aimed to give deeper insight into the impact of the Covid-19 pandemic on mental health (i.e., resilience and well-being) and the functioning of support structures in the vulnerable population of multi-problem families, by testing three hypotheses: (1) pandemic-related stress is negatively associated with youth and parental mental health; (2) experienced support is positively associated with youth and parental mental health; and (3) experienced support is a protective factor minimizing the negative effects of pandemic-related stress on youth and parental mental health. Results showed that youth experiencing higher levels of pandemic-related stress reported lower levels of well-being, irrespective of perceived informal or formal support. Pandemic-related stress was also associated with youth resilience, yet only for youth reporting low levels of perceived formal support. Furthermore, perceived support was positively associated to mental health in both youth and parents from multi-problem families, yet did not further moderate the effect of pandemic-related stress on mental health.

Despite our expectations, pandemic-related stress was not consistently associated with mental health of youth and parents from multi-problem families, with one exception: Higher levels of pandemic-related stress were related to lower levels of youth well-being. This demonstrates that Covid-19 pandemic-related stress has not systematically negatively affected the mental health of multi-problem families, and parents in particular. Perhaps pandemic-related stress did not impact their mental health, as we measured mental health as two broad constructs which were not directly impacted by the pandemic. For example, it is arguable that resilience, which was measured as the individual and environmental resources available to participants [44], were not immediately lost as a result of pandemic-related stress. This could also suggests that the common assumption that high-risk groups, such as multi-problem families, are vulnerable in stressful situations [6, 7], may be inadequate. That is, individual resiliency and good mental health depend not only on the history of adversity and environmental risk factors, but also on individual strengths, including intelligence and personality [56]. Furthermore, some people with a history of adversity might be even less affected by recent stressors, such as pandemic-related stress, as they have learned to cope with adversity [57]. This could suggest that multi-problem families, who have experienced adversity, might have developed coping styles that proved useful to deal with the challenges during the Covid-19 pandemic, thereby reducing the negative impact of pandemic-related stress on mental health. Possibly, the adequate coping styles may have also kept stress levels low. In fact, the average levels of pandemic-related stress were quite low in both youth and parents (see Table 2), suggesting that these families had a certain flexibility to cope with the pandemic without experiencing a lot of stress and subsequent mental health consequences.

In line with our expectation, youth, however, did report some (elevated) mental health problems when experiencing pandemic-related stress, especially if they had no or weak therapist support. This suggests that the negative effects of pandemic-related stress may be stronger for youth than adults. That is, youth may be more effected by the pandemic and the imposed measures as social activities are particularly important during adolescence, while youth are less susceptible to severe Covid-19 infections [8, 9]. Our findings also showed that perceived pandemic-related stress was a better predictor of youth mental health than the duration of the pandemic and the actual imposed restrictions.

The current study also demonstrated that support was related to higher levels of mental health in both youth and parents of multi-problem families. This shows that support is indeed an important factor for promoting mental health, also during the Covid-19 pandemic [2, 25, 30]. More specifically, this study showed that when youth have an informal mentor and when the quality of the mentoring relationship was perceived as high, these youth reported higher levels of resilience and well-being. In line with previous research, this indicates that informal mentoring relationships can have beneficial effects for youth [26, 27, 30]. Additionally, we showed that the therapeutic alliance was positively associated with youth resilience and well-being, whereas the mere presence of a therapist was not. In parents, however, both the presence of a therapist and a strong therapeutic alliance were linked to high levels of resilience and well-being. Similar to previous findings [36, 58], our results suggest that the therapeutic alliance as perceived by clients is an important factor to consider in mental health care.

We found little evidence that support protected youth and parents from the negative effects of pandemic-related stress on mental health. The direct effects of experienced support on mental health, but lack of interaction effects suggest that support plays a compensatory rather than protective role in mental health [59], and is therefore still an important factor in promoting mental health. Yet, we found one significant interaction effect in youth: A strong therapeutic alliance protected youth from a negative effect of pandemic-related stress on resilience. That is, the negative effect of pandemic-related stress on resilience only existed for youth not receiving therapy or perceiving the therapeutic alliance as relatively weak. This demonstrates that therapeutic alliance is a key factor in mental health care that can not only improve mental health directly, but can also buffer against additional stressors during the treatment process, which is in line with previous research [33, 34].

### Implications

The results of the current study can inform policy makers and mental health care professionals about the mental health and support structures of multi-problem families during a pandemic. The findings are promising, as they show that individuals may not be as severely affected by Covid-19 pandemic-related stress as we expected, even in the presence of pre-pandemic risk factors, as is the case with multi-problem families [4, 6, 21]. Yet, cautious optimism is advised given that we found associations between pandemic-related stress and youth mental health. That is, youth who experience pandemic-related stress are more likely to experience low levels of well-being and—if the therapeutic alliance is weak—lower resilience. Good mental health care is therefore essential for youth of multi-problem families who experience pandemic-related stress, or else these youth risk (elevated) mental health problems.

Our results also demonstrated that perceived support was positively associated with mental health, which stresses the need to support youth and parents by strengthening their informal and formal networks. That is, our study suggests that individuals in need could benefit from professional help (i.e., presence of a therapist and a strong therapeutic alliance) and, in the case of youth, informal support (i.e., the presence of an informal mentor and a high mentor relationship quality). Strengthening the therapeutic alliance is even more important in youth, as a strong therapeutic alliance protects youth from negative consequences of pandemic-related stress on resilience. Furthermore, since the mere presence of an informal mentor is associated with higher levels of youth mental health, it is important to help youth in finding supportive non-parental adults, for example through youth-initiated mentoring [40, 41] or social capital interventions [60].

### Strengths and limitations

This study is unique in several respects. First, we sampled a hard-to-reach population, namely that of multi-problem families, which is quite rare for research in general and, to our knowledge, our study was the first on mental health during the Covid-19 pandemic in this population. Second, most participants reported on multiple measurements during the pandemic, giving us insight into the links between pandemic-related stress, support and mental health in different phases of the pandemic, both during lockdowns and in times with very few restrictions, giving us more certainty of the robustness of our results.

This study also has limitations. First, we did not include pre-pandemic measurements, so it is unknown whether mental health changed as a result of the pandemic. Second, we only investigated the relation between pandemic-related stress and mental health. We have no knowledge on whether other aspects or consequences of the Covid-19 pandemic may have influenced the mental health of individuals (e.g., experienced loss of loved ones due to Covid-19) [22]. However, we also included pandemic duration and pandemic severity as covariates, which did not correlate significantly to mental health (see Table 3). Third, despite our efforts, the sample size is rather small for the number of associations tested, thus, our results should be interpreted carefully. Fourth and finally, parents did not report on informal support, thereby restricting the possibility to examine both types of support in parents. Future research could examine whether informal mentoring relates to parental mental health, as it does for youth.

## Conclusion

In sum, this study demonstrated that youth from multi-problem families are at risk for mental health problems when experiencing pandemic-related stress, while parental mental health was not negatively effected by pandemic-related stress. Youth and parents who experienced support during the pandemic reported higher levels of resilience and well-being, showing that offering support is important to promote mental health during the pandemic. Our findings further demonstrate the importance of the therapeutic alliance in mental health care for both youth and parents [33, 34], and the potential of informal mentoring for improving youth mental health [26, 40].

## Supplementary Information

**Additional file 1:** **Table S1.** Pandemic-related stress questionnaire. **Table S2.** Active measures by the Dutch Government during risk levels.

## Data Availability

The dataset analyzed during the current study is accessible by all authors. Access was also granted to students or research assistants who assisted in data collection for the duration of their research project membership, after signing a confidentiality agreement. The dataset is not publicly available due to sensitivity and confidentiality of data, but is available from the corresponding author on reasonable request after completion of the research project.
