# Supplementary material for: Resilience, well-being and informal and formal support in multi-problem families during the Covid-19 pandemic
Source: Child Adolesc Psychiatry Ment Health. 2022 Dec 19;16:103. doi: 10.1186/s13034-022-00542-2 (PMC9762621; doi:10.1186/s13034-022-00542-2)
Supplement: Supplementary file 1 — Additional file 1: Table S1. Pandemic-related stress questionnaire. Table S2. Active measures by the Dutch Government during risk levels. [file 13034_2022_542_MOESM1_ESM.docx]

**Additional File 1**

**Additional Tables**

**Table S1**

*Pandemic-related stress questionnaire*

| Items |
| --- |
| 1. I'm afraid my family will be infected with the coronavirus. |
| 2. The coronavirus crisis leads to money problems for me and/or my family. |
| 3. I'm having a great time now that I'm home more because of the coronavirus crisis. |
| 4. I'm afraid that my education will be delayed due to the coronavirus crisis. |
| 5. Due to the coronavirus crisis, I am less able to share my concerns with others. |
| 6. Due to the coronavirus crisis, I often argue with my family members. |
| 7. I am afraid that I will be infected with the coronavirus. |
| 8. Due to the coronavirus crisis I am worried about my future. |
| 9. My life has become a lot more boring due to the coronavirus crisis. |
| 10. Due to the coronavirus crisis I find it scary to be around other people. |
| 11. Due to the coronavirus crisis I feel lonely. |
| 12. I try to take care of others in this coronavirus time. |

*Note.* Item 4 was only administered to youth.

**Table S2**

*Active Measures by the Dutch Government During Risk Levels*

|  | 1. Vigilant | 2. Worrisome | 3. Serious | 4. Very serious |
| --- | --- | --- | --- | --- |
| Quarantine | Yes | Yes | Yes | Yes |
| No. of people visiting per day (excl. children <12 y.o.) | ≤8 | ≤6 | ≤4 | ≤2 |
| Groups in public places (excl. children <12 y.o.) | ≤8 | ≤6 | ≤4 | ≤2 |
| Working from home | Yes | Yes | Yes | Yes |
| Curfew | No | No | No | Possible 21:00-04:30 |
| Restaurants | - Mandatory reservation and health check - ≤8 people per table - ≤100 guests inside | - Mandatory reservation and health check - ≤6 people per table - ≤50 guests inside | Closed | Closed |
| Clubs | Closed | Closed | Closed | Closed |
| Supermarkets | Open | Open to vulnerable groups at special times | Open to vulnerable groups at special times | Open to vulnerable groups at special times |
| Other shops | Open | Open | Open | Closure of non-essential retail outlets possible |
| Sports | ≤100 people as audience | - No audience - Dressing rooms and showers closed | - No audience - Dressing rooms, showers and canteen closed | - No audience - Dressing rooms, showers and canteen closed - Sports practice at 1.5m distance; with ≤2 people (except for children <17 y.o.) - Possible to forbid sporting indoors |
| Events | - Mandatory reservation and health check - ≤100 guests | - Mandatory reservation and health check - ≤50 guests | - Events prohibited | - Events prohibited |
| School & daycare | Open | Open | Open | Possible to limit physical education in primary, secondary and higher education |

*Note.* Adapted from <https://www.rijksoverheid.nl/binaries/rijksoverheid/documenten/publicaties/2021/04/13/routekaart-coronamaatregelen/routekaart-coronamaatregelen.pdf>. Copyright 2021 by Central Government of the Netherlands.
